# Isometric versus isotonic contractions: Sex differences in the fatigability and recovery of isometric strength and high‐velocity contractile parameters

**DOI:** 10.14814/phy2.14821

**Published:** 2021-05-15

**Authors:** Phuong L. Ha, Benjamin E. Dalton, Michaela G. Alesi, Tyler M. Smith, Trisha A. VanDusseldorp, Yuri Feito, Garrett M. Hester

**Affiliations:** ^1^ Department of Exercise Science and Sport Management Kennesaw State University Kennesaw GA USA; ^2^ Department of Molecular and Cellular Biology Kennesaw State University Kennesaw GA USA

**Keywords:** fatigue, plantar flexors, power, rate of muscle activation, skeletal muscle

## Abstract

The purpose of this study was to investigate potential sex differences in the fatigue‐ and recovery‐induced responses of isometric strength and power, as well as select dynamic contractile parameters after isometric and isotonic plantar flexor (PF) contractions. Healthy males (*n* = 12; age = 21.8 ± 2.2 years) and females (*n* = 14; age = 21.4 ± 2.5 years) performed a 2‐min maximal voluntary isometric contraction and 120 concentric isotonic (30% peak isometric torque) contractions of the PFs on separate visits. Isometric strength, isotonic power, as well as torque‐ and velocity‐related parameters were recorded before, immediately after, and throughout 10 min of recovery. Rate of EMG rise (RER) for the medial gastrocnemius (MG) and soleus was also obtained. All measures responded similarly between sexes after both fatiguing modalities (*p* > 0.05), except RER of the MG which, in males demonstrated both, a greater decrease during isotonic contractions (*p* = 0.038, $\eta_{p}^{2}$ = 0.174) and more rapid recovery after isometric exercise (*p* = 0.043, $\eta_{p}^{2}$ = 0.166). Although not significant, a nearly large effect size was demonstrated for the fatigue‐induced decrease in isometric strength (*p* = 0.061; *d* = 0.77) due to relative decreases tending to be greater in males (−29% vs. −17%). Regardless of fatiguing modality, sex differences were minimal for fatigue and recovery‐related responses in muscle function for the PFs, although the difference for RER may indicate a unique origin of fatigue. Further support for the disassociation between the response in isometric strength and power after fatiguing exercise was also demonstrated.

## Graphical abstract

Fatigue‐induced decrements in isometric strength tend to be attenuated in females compared to males, but less is clear for isotonic power and the plantar flexors have seldomly been examined. We show that fatigability and recovery of strength and power were similar in male and females after isotonic and isometric exercise. This was despite the unique responses in rapid muscle activation between sexes.

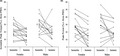

## 1 INTRODUCTION

Performance fatigue can be defined as an exercise‐induced decrease in the maximal force or power generated by skeletal muscle (Bigland‐Ritchie et al., 1978; Enoka & Duchateau, 2008). Previous research has focused on isometric strength as an indicator of performance fatigue likely due to the relative ease of measurement and ability to incorporate physiological assessments (e.g., peripheral nerve stimulation). There is far less evidence for the fatigue‐ and recovery‐related response of power. Further, there are divergent decrements in power and isometric strength after fatiguing isotonic contractions (Akagi et al., 2020; Cheng & Rice, 2005; Krüger et al., 2019). It was recently shown that the fatigue‐induced reduction in isometric strength was not associated with the reduction in power during isotonic contractions (Akagi et al., 2020). Thus, it is warranted to investigate power and isometric strength as standalone performance measures to better understand fatigue‐ and recovery‐related sex differences in muscle function.

Fatigue‐induced decrements in isometric strength tend to be less in females (Avin et al., 2010; Clark et al., 2003; Hunter et al., 2006), yet this appears to be dependent upon the contraction type (Clark et al., 2003), intensity (Hunter, 2016a) and muscle group (Avin et al., 2010). In particular, less is clear regarding sex differences in fatigability after dynamic contractions (Hunter, 2016b). Most studies directly comparing sex differences in fatigue between isometric and dynamic muscle contractions have only assessed maximal strength (Clark et al., 2003; Maughan et al., 1986). Furthermore, to the best of our knowledge, only one study has compared sex differences in fatigue‐ *and* recovery‐related responses induced by isometric and isotonic muscle contractions, though power was only assessed during and after the isotonic protocol (Senefeld et al., 2018). Senefeld et al. (2018) determined that fatigue‐induced decreases in isometric strength of the knee extensors was greater in males only after isotonic contractions, while the response in power was similar between sexes after isotonic fatigue. However, recovery of isometric strength was quicker in females after both modalities, while power recovered similarly. In contrast to Senefeld et al. (2018) and Lanning et al. (2017) reported greater fatigue‐induced decrements in power for males during isotonic contractions of the plantar flexors (PFs), but recovery was not examined (Lanning et al., 2017). These findings highlight that the PFs may demonstrate a unique response compared to the more commonly investigated knee extensors. Research investigating both isometric and dynamic fatiguing exercise is needed to yield further insight related to the influence of contraction type on sex differences in fatigability and recovery, in particular with the response in power. An improved understanding of sex differences in the fatigability and recovery of muscle function should be helpful towards improving exercise prescription as it can be tailored for the understudied female population.

Independently assessing torque and velocity during isotonic contractions provides an opportunity to understand the contribution of these mechanical factors to decrements in power during and after (i.e., recovery) fatigue. For example, a fatigue‐induced change in the position at which peak power occurs (Cheng & Rice, 2005), which would likely coincide with an altered muscle length–tension relationship and should influence the torque and velocity corresponding with peak power (Cormie et al., 2011). It is also important to consider other parameters that are particularly time‐dependent, such as rate of torque development (RTD), which was recently identified as a predictor of isotonic power (Olmos et al., 2019), as well as rate of velocity development (RVD) since peak power tends to occur prior to peak velocity. The maintenance of rapid muscle activation (i.e., rate of electromyography rise; RER) is likely influential for maintaining power during and after fatigue (Wallace et al., 2016), yet its response may differ between sexes after isometric and isotonic exercise due to unique physiological impairments (Senefeld et al., 2018). For example, Senefeld et al. (2018) showed that males demonstrated greater supraspinal fatigue during and after isometric fatiguing exercise alone. Given the mixed muscle fiber type composition of the PFs (Johnson et al., 1973), the response of time‐dependent measures to isometric and isotonic fatigue may be different for the PFs compared to more commonly assessed muscles such as the knee extensors and elbow flexors. Despite the important role of the PFs for locomotion (McGibbon, 2003), the PFs are an understudied muscle group as it relates to sex differences in fatigue (Hunter, 2018; see Figure 3). Thus, the purpose of this study was to investigate potential sex differences in the fatigue‐ and recovery‐induced responses of isometric strength and power, as well as select dynamic contractile parameters after isometric and isotonic PF contractions. We hypothesized that the decrease in isometric strength will be greater in males than females *only* after a sustained isometric contraction. In addition, males will exhibit a slower rate of recovery for isometric strength after a sustained isometric contraction. In contrast to isometric exercise, we hypothesized that strength, power, and select dynamic contractile parameters will decrease and recover similarly between sexes after fatiguing isotonic contractions.

## 2 MATERIALS AND METHODS

### 2.1 Participants

Based on the findings for isotonic power from Lanning et al. (2017), an a priori analysis (G*Power v. 3.0.10) was used to estimate that a total sample size of 26 was needed for an independent samples *t*‐test to provide a statistical power of 0.83. Twenty‐seven healthy, males (*n* = 13) and females (*n* = 14) who reported having performed 1–3 days/week of lower‐body resistance training and endurance exercise over the past 6 months volunteered for this study. Due to software issues, data collection was unable to be completed for one male participant thus only 12 males completed the study. Individuals were excluded if they reported being a student‐athlete or club sports athlete, running more than 60 min per week, supplementing with beta‐alanine or creatine, or having any disease or illness. In addition, individuals were excluded if they had a body mass index over 30 kg·m^−2^, were not cleared for physical activity via the Physical Activity Readiness Questionnaire or suffered a leg injury within the past year. Pregnant females were excluded and only those who reported taking the same oral contraceptive for at least 6 months were included. In addition, both testing visits were performed during the first 2 weeks of the oral contraceptive consumption phase for females. A 24‐h dietary recall was provided prior to each testing visit in order to calculate carbohydrate and total calorie intake using the myfitnesspal application (Version 20.7.0.29453). Participants were instructed to arrive after a 4 h fast prior to the first visit only. Finally, participants were instructed to avoid alcohol as well as endurance and resistance exercise for 24 and 48 h, respectively, prior to all visits. Prior to data collection, this study was approved by the Institutional Review Board under the Ethical Principles and Guidelines for the Protection of Human Subjects of Research report. All participants provided oral and written consent prior to beginning the study.

### 2.2 Experimental design

This cross‐over designed study consisted of participants visiting the laboratory on three separate occasions, separated by 2–7 days. The first visit consisted of all paperwork and surveys, body composition testing, and familiarization with PF testing and fatigue protocols. Participants performed either the isometric or isotonic PF fatigue protocol and corresponding testing on visits 2 and 3. The order in which the fatigue protocols were completed was randomly assigned prior to the first visit.

Torque, velocity, and ankle joint position were recorded during concentric PF contractions of the dominant leg using a calibrated Biodex 4 isokinetic dynamometer (Biodex Medical Systems, Inc.). These signals and electromyography (EMG) were sampled at 2 kHz using an 8‐channel Bagnoli Desktop System (Delsys, Inc.). EMG of the medial gastrocnemius (MG) and soleus were recorded using parallel bar, bipolar surface electrodes (Delsys Bagnoli, Delsys, Inc.). A reference electrode was placed over the C7 vertebrae. Prior to electrode placement, the skin was shaved, abraded, and cleaned with alcohol, and subsequently, the electrodes were applied in accordance with the SENIAM project recommendations (Hermens, 1997). Participants were seated with hands across the chest, restraining straps over the trunk and pelvis, and the input axis of the dynamometer aligned with the axis of rotation of the ankle. The knee was extended to ~180° and hip was maintained at a comfortable angle (Dalton et al., 2014). The foot was secured to the footplate with two straps over the dorsal aspect and a custom ankle wrap technique using a non‐elastic wrap to anchor the heel to the footplate. Ankle position was set at a neutral angle (neutral = 90°) for isometric contractions and at the beginning of isotonic contractions, which involved a 25° range of motion (ROM); 0°–25° plantar flexion). EMG of the vastus lateralis was monitored to aid in preventing contribution of the knee extensors during testing. All participants received consistent, *strong* verbal encouragement and visual feedback during the testing and fatigue protocols.

#### 2.2.1 Body composition

Height (cm) and weight (kg) were measured using an electronic physician scale (Tanita WB 3000). Total body fat % was obtained via bioelectrical impedance analysis (InBody770, InBody Co.) following manufacturer recommendations.

#### 2.2.2 Baseline testing

Prior to testing, participants performed two submaximal isometric PF contractions at 50% and 75% of perceived maximal effort. Participants then performed three, 3‐ to 5‐s maximal voluntary isometric contractions (MVICs) separated by 1 min of rest. Additional MVIC trials were performed if peak torque from the first three trials varied by more than 5%. Participants then performed five isotonic practice trials in preparation for isotonic testing. Subsequently, five maximal isotonic PF contractions were performed to obtain baseline (i.e., pre) values. All isotonic contractions were performed at 30% of the MVIC peak torque. Participants were instructed to stay completely relaxed as investigators reset the level arm to a 90° joint angle in between repetitions. In addition, participants were instructed to push “as hard and fast as possible” for isotonic contractions, whereas “as hard as possible” were the instructions for MVICs.

#### 2.2.3 Isometric and isotonic fatigue protocols

The isometric fatigue protocol consisted of a 2‐min sustained MVIC. The dynamic fatigue protocol required the performance of 120 maximal PF isotonic contractions at 30% MVIC with ~1.5–2 s separating contractions (Lanning et al., 2017). A single MVIC followed by five maximal isotonic PF contractions was performed immediately (POST‐IMM; ~6 s), 2.5 (POST‐2.5), 5 (POST‐5), and 10 min (POST‐10) after both fatigue protocols. RPE (Category Ratio Scale‐10) (Neely et al., 1992) was recorded at POST‐IMM as well as each recovery timepoint.

#### 2.2.4 Data analysis

**FIGURE 1:**
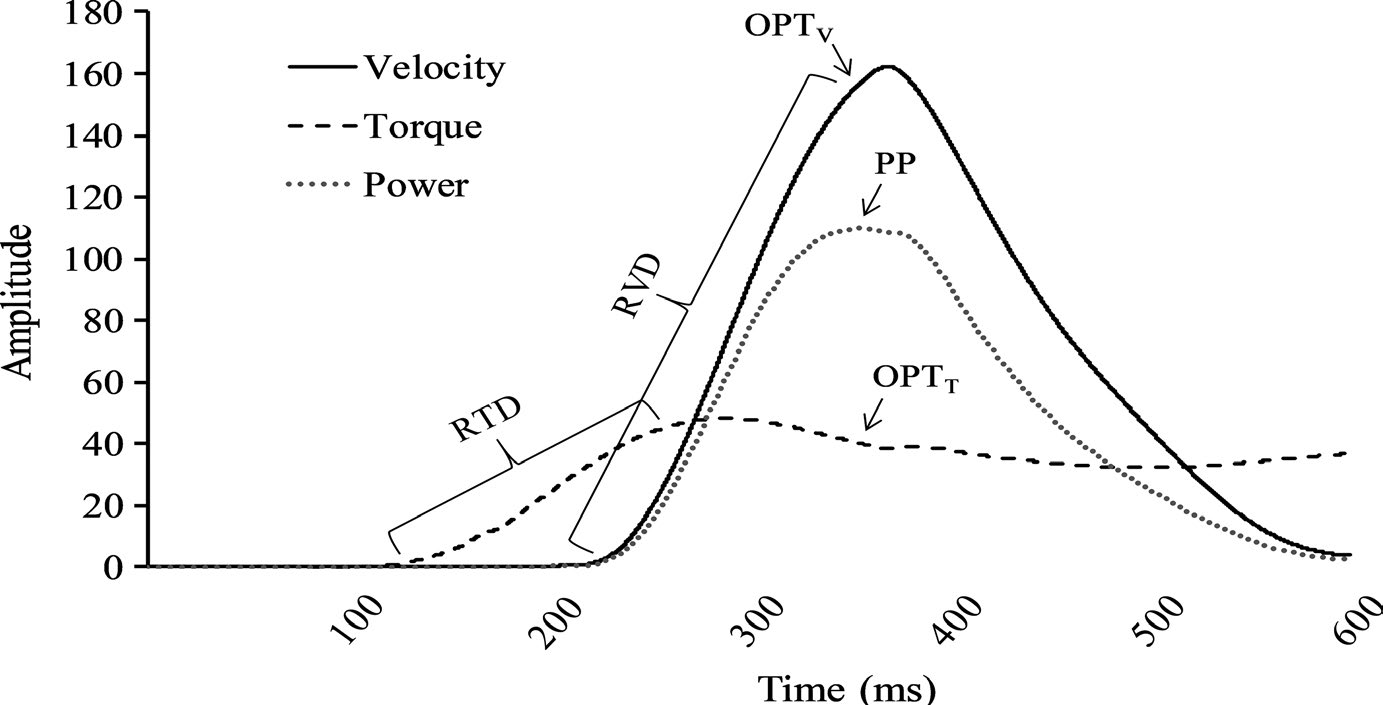
Example of the processed isotonic signals and the dependent variables (OPT_T_, optimal torque; OPT_V_, optimal velocity; PP, peak power; RTD, rate of torque development; RVD, rate of velocity development) that were recorded.

All data analysis was performed using custom written software (LabVIEW, National Instruments). Isometric peak torque (i.e., isometric strength) was considered the highest 500 ms average during MVICs. For isotonic contractions, the velocity signal was converted to radians and multiplied by torque to calculate power and the peak power was recorded. In addition, velocity and torque at the moment in time peak power occurred were recorded as optimal velocity (OPT_V_) and torque (OPT_T_), respectively (Lanning et al., 2017). RTD and RVD were determined as the maximum linear slope value (Δtorque/Δtime and Δvelocity/Δtime, respectively) calculated over a 20 ms rolling average until the time at which peak power occurred (Wallace et al., 2016). ROM was calculated by subtracting the starting joint position from the end position, and the position at peak power (POS_PP_) was recorded. An example of the processed isotonic signals and the dependent variables recorded is displayed in Figure 1. EMG was recorded during the isotonic contractions and subsequently, processed using a fourth‐order Butterworth filter with a low‐ and high‐frequency cutoff of 20 and 400 Hz, respectively, which was applied to the scaled zero means EMG signal. The signal was then smoothed using a zero‐lag, low pass filter (10 Hz) and normalized to its peak amplitude (PEMG). RER was obtained from the linear slope of the normalized EMG signal and calculated as the highest rolling 50 ms value (%PEMG·s^−1^) up to peak power for the MG (RER_MG_) and soleus (RER_SOL_) Thompson et al., 2014; Van Driessche et al., 2019). The average of the previously described dependent variables was calculated for contractions 1–5, 56–60, and 116–120 from the fatiguing isotonic protocol and the 5 isotonic contractions performed at each timepoint (e.g., baseline, POST‐IMM, POST‐2.5, etc.). The averages were used for statistical analysis, after being converted to percentages, as described in the following section.

### 2.3 Statistical analyses

Independent samples *t*‐tests were used to compare characteristics between sexes, whereas dependent samples *t*‐tests were used to compare carbohydrate and total daily calorie intake between visits. Two‐way (sex × contraction type) repeated measures analyses of variance (ANOVAs) were used to compare baseline performance data between sexes for both protocols. For each dependent variable, relative change for *fatigue* was calculated as [(end value–baseline value)/baseline value] × 100, whereas [(end value−POST‐IMM)/POST‐IMM] × 100 was calculated to determine relative change during *recovery* (Senefeld et al., 2013; Senefeld et al., 2018). Separate analyses were used on the relative change values to assess fatigue and recovery of the following dependent variables: peak power, isometric peak torque, OPT_V_, OPT_T_, RVD, RTD, ROM, POS_PP_, RER_MG_, RER_SOL_, and RPE. Two‐way (sex × time [contractions 56–60 vs. 116–120]) repeated measures ANOVAs were used to assess fatigue *during* the dynamic protocol. Independent samples *t*‐tests and two‐way (sex × time) repeated measures ANOVAs were used to compare fatigue‐ and recovery‐induced changes, respectively, for each protocol. Specifically, relative change at POST‐IMM was used to examine fatigue, while relative changes at POST‐2.5, POST‐5, and POST‐10 were compared to assess recovery. Post hoc analyses with a Bonferroni correction factor were used to identify differences between timepoints when appropriate. Pearson product‐moment correlation coefficients were calculated to examine the association between fatigue‐ and recovery‐related changes in isometric strength and power, as well as correlations between dynamic contractile parameters and power. Statistical analyses were performed using PASW version 27.0 (SPSS Inc) and an α level of *p* ≤ 0.05 was used to determine statistical significance. EMG data for one male participant were missing due to technical issues during data collection, thus a sample size of 11 was used for this group during analyses of EMG variables. Effect sizes were reported using partial eta squared ($\eta_{p}^{2}$) for ANOVA analyses and <0.06, 0.07−0.14, and >0.14 indicated small, medium, and large effect sizes, whereas Cohen's *d* was used for pairwise comparisons with 0.30, 0.50, and 0.80 indicating the same effect size classifications (Cohen, 1988). Data is provided as mean ±SD in the text and tables, while mean ± SEM is used in figures. Select figures were constructed using the templates based on Weissgerber et al. (2015).

## 3 RESULTS

### 3.1 Baseline measurements

Characteristics for the male and female group are displayed in Table 1. Males were taller (*p* < 0.001, *d* = 2.47), possessed greater body mass (*p* < 0.001, *d* = 2.48), and had a lower body fat% (*p* < 0.001, *d* = 1.90). There were no differences in age (*p* = 0.736, *d* = 0.13), aerobic exercise (*p* = 0.605, *d* = 0.21), or lower body resistance training (*p* = 0.563, *d* = 0.24) between sexes.

**TABLE 1:** Group characteristics

Variable	Males	Females	% Difference
Age (y)	21.7 ± 2.2	21.4 ± 2.5	1.47%
Height (cm)	173.5 ± 4.0	160.5 ± 6.3*	7.51%
Body Mass (kg)	78.6 ± 8.7	58.5 ± 7.3*	25.57%
BMI (kg·m^−2^)	26.1 ± 2.6	22.6 ± 2.0*	13.14%
Body Fat%	19.0 ± 6.9	30.8 ± 5.5*	38.45%
Aerobic (h/wk)	2.1 ± 1.6	2.5 ± 1.7	13.89%
Lower Body RT (h/wk)	2.1 ± 0.8	2.0 ± 0.5	7.83%

Abbreviations: BMI, Body mass index; METs, metabolic equivalent of task; RT, resistance training.

* Significant (*p* < 0.05) difference between sexes.

Baseline performance data for the isometric and isotonic fatigue visit is provided in Tables 2 and 3, respectively. No sex × contraction type interactions were present for any measures (*p* > 0.05). A main effect for contraction type was present for power (*p* = 0.022, $\eta_{p}^{2}$ = 0.20; isometric = 196.72 ± 54.80 W vs. dynamic = 183.82 ± 50.23 W) as it was higher at baseline during the isometric protocol, whereas all other dependent variables were similar between protocols (*p* > 0.05). Power (*p* < 0.001, $\eta_{p}^{2}$ = 0.52), isometric peak torque (*p* = 0.001, $\eta_{p}^{2}$ = 0.392), OPT_V_ (*p* < 0.001, $\eta_{p}^{2}$ = 0.49), OPT_T_ (*p* < 0.001, $\eta_{p}^{2}$ = 0.43), RVD (*p* < 0.001, $\eta_{p}^{2}$ = 0.43), RTD (*p* < 0.001, $\eta_{p}^{2}$ = 0.59), RER_MG_ (*p* = 0.005, $\eta_{p}^{2}$ = 0.29), and RER_SOL_ (*p* < 0.001, $\eta_{p}^{2}$ = 0.57) were greater in males. Self‐reported carbohydrate intake (178.46 ± 84.78 g vs. 177.50 ± 65.93 g) and total calories (1,643.23 ± 1,006.04 kcal vs. 1,560.58 ± 530.83 kcal) were similar between visits (*p* = 0.955, *d* = 0.01 and *p* = 0.597, *d* = 0.10, respectively).

**TABLE 2:** Absolute and relative change values for the isometric fatigue visit

	Males			Females			*p*‐value*	*d* *
	PRE	POST	Relative change	PRE	POST	Relative Change		
ISOM_TQ_	161.47 (35.05)	114.91 (34.90)	−29.42 (11.45)	113.67 (26.40)	90.96 (17.30)	−17.01 (18.51)	0.061	0.77
PP	239.24 (44.27)	167.91 (43.33)	−28.96 (17.17)	160.28 (31.93)	115.81 (25.39)	−26.94 (14.94)	0.752	0.12
OPT_V_	200.72 (14.80)	170.10 (12.60)	−14.78 (9.52)	180.79 (10.99)	159.26 (19.25)	−11.94 (8.43)	0.428	0.31
OPT_T_	68.23 (11.30)	56.14 (12.28)	−17.75 (11.57)	50.77 (9.29)	41.49 (7.59)	−17.73 (9.73)	0.996	0.00
RVD	2492.47 (458.98)	1858.13 (187.10)	−23.46 (14.87)	2044.53 (295.69)	1600.26 (448.59)	−22.07 (14.86)	0.814	0.09
RTD	864.40 (214.98)	737.27 (130. 04)	−11.14 (20.90)	566.72 (125.47)	537.10 (178.26)	−3.81 (28.58)	0.469	0.28
RER_MG_	1210.04 (236.40)	1247.92 (178.18)	5.85 (20.22)	1142.05 (178.29)	1054.38 (192.10)	−6.91 (14.99)	0.083	0.73
RER_SOL_	1296.95 (159.24)	1273.60 (143.47)	0.15 (21.32)	1083.22 (149.38)	1012.52 (155.92)	−5.12 (18.77)	0.517	0.26
ROM	24.55 (0.47)	23.98 (0.97)	−2.35 (2.71)	24.50 (0.46)	24.06 (0.71)	−1.79 (1.76)	0.536	0.24
POS_PP_	15.29 (2.48)	14.30 (2.54)	−6.44 (6.00)	14.85 (2.19)	14.44 (2.40)	−2.95 (3.73)	0.099	0.71

Abbreviations: AVG_TQ_, average torque;ISOM_TQ_, Isometric peak torque; OPT_T_, optimal torque; OPT_V_, Optimal velocity; POS_PP_, position at peak power; PP, Peak power; RER_MG_, medial gastrocnemius rate of electromyography rise; RER_SOL_, soleus rate of electromyography rise; ROM, range of motion; RTD, rate of torque development; RVD, rate of velocity development.

* Based on analysis of relative change values.

**TABLE 3:** Absolute and relative change values for the isotonic fatigue visit

	Males			Females			*p*‐value*	*d* *
	PRE	POST	Relative Change	PRE	POST	Relative Change		
ISOM_TQ_	155.22 (33.71)	125.07 (34.46)	−19.42 (12.46)	114.94 (21.64)	97.89 (18.56)	−13.80 (10.95)	0.216	0.50
PP	218.80 (45.88)	175.46 (37.57)	−18.71 (13.87)	153.84 (31.21)	129.36 (27.94)	−15.25 (13.96)	0.533	0.24
OPT_V_	193.74 (18.60)	176.49 (16.22)	−8.64 (6.43)	173.14 (12.16)	161.02 (11.42)	−6.83 (5.78)	0.457	0.31
OPT_T_	64.35 (10.46)	56.76 (10.13)	−11.58 (9.23)	50.60 (7.37)	45.63 (7.96)	−9.73 (10.03)	0.632	0.00
RVD	2361.02 (455.67)	1975.22 (327.07)	−14.94 (13.79)	1841.02 (275.66)	1605.47 (254.21)	−12.09 (12.05)	0.580	0.09
RTD	812.51 (138.65)	703.67 (137.07)	−12.51 (14.99)	526.93 (109.37)	488.23 (116.84)	−6.07 (19.75)	0.365	0.28
RER_MG_	1321.03 (135.41)	1131.43 (157.02)	−14.48 (7.04)	1100.98 (121.95)	971.25 (151.52)	−10.55 (18.68)	0.478	0.73
RER_SOL_	1337.83 (137.59)	1158.51 (253.10)	−13.73 (15.05)	1089.74 (114.86)	998.19 (90.02)	−7.22 (14.65)	0.287	0.26
ROM	24.20 (0.58)	24.13 (0.42)	−0.26 (1.54)	24.63 (0.45)	24.60 (0.49)	−0.14 (0.99)	0.813	0.24
POS_PP_	14.37 (1.94)	14.01 (2.07)	−2.55 (4.17)	14.98 (2.15)	14.86 (2.21)	−0.85 (2.37)	0.205	0.71

Abbreviations: AVG_TQ_, average torque;ISOM_TQ_, Isometric peak torque; OPT_T_, optimal torque; OPT_V_, Optimal velocity; POS_PP_, position at peak power; PP, Peak power; RER_MG_, medial gastrocnemius rate of electromyography rise; RER_SOL_, soleus rate of electromyography rise; ROM, range of motion; RTD, rate of torque development; RVD, rate of velocity development.

* Based on analysis of relative change values.

### 3.2 Fatigue during isotonic contractions

**FIGURE 2:**
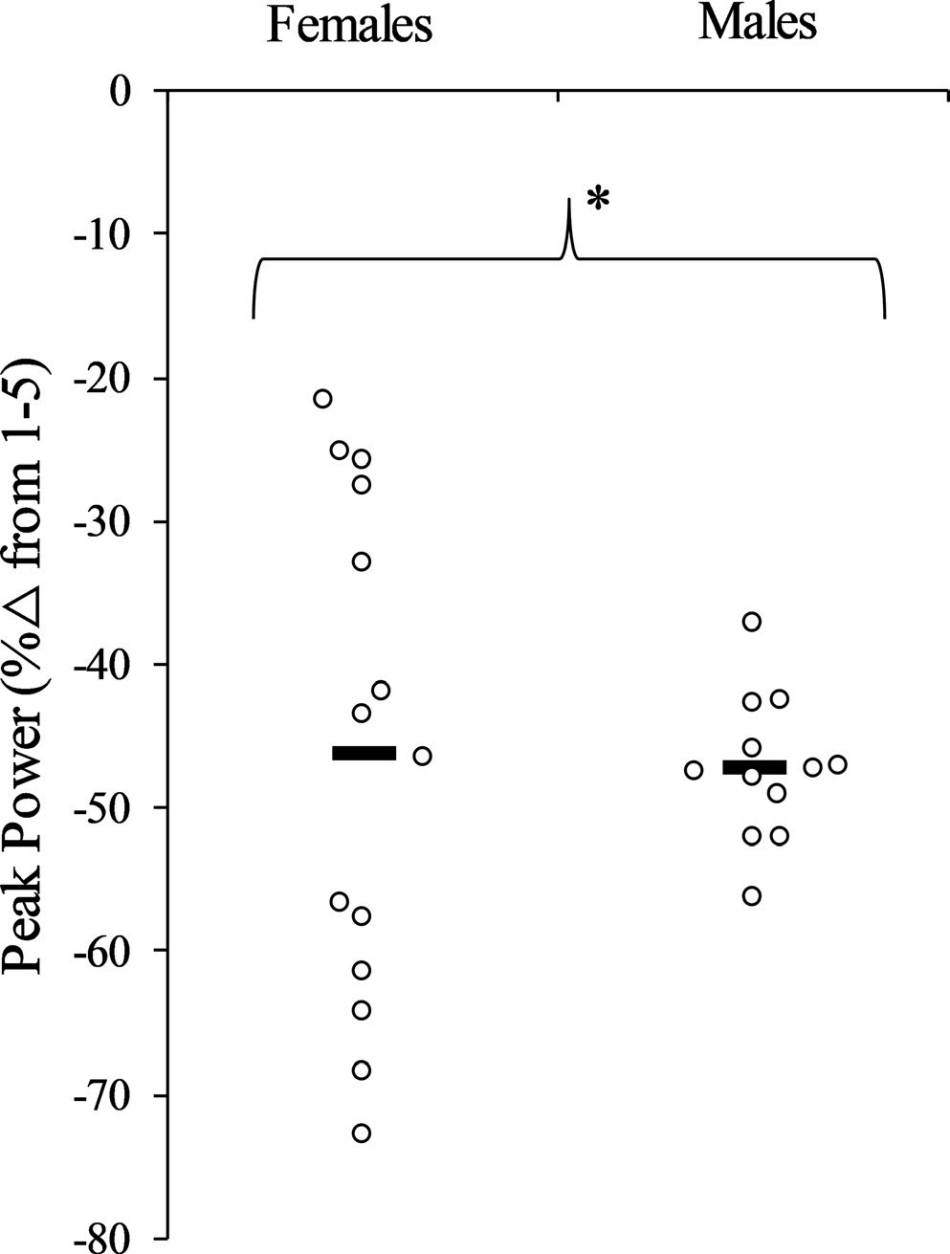
Individual responses and group means (bars) for peak power during contractions 116–120 of the isotonic fatigue protocol. * significant (*p* < 0.001) decrease, regardless of sex

**FIGURE 3:**
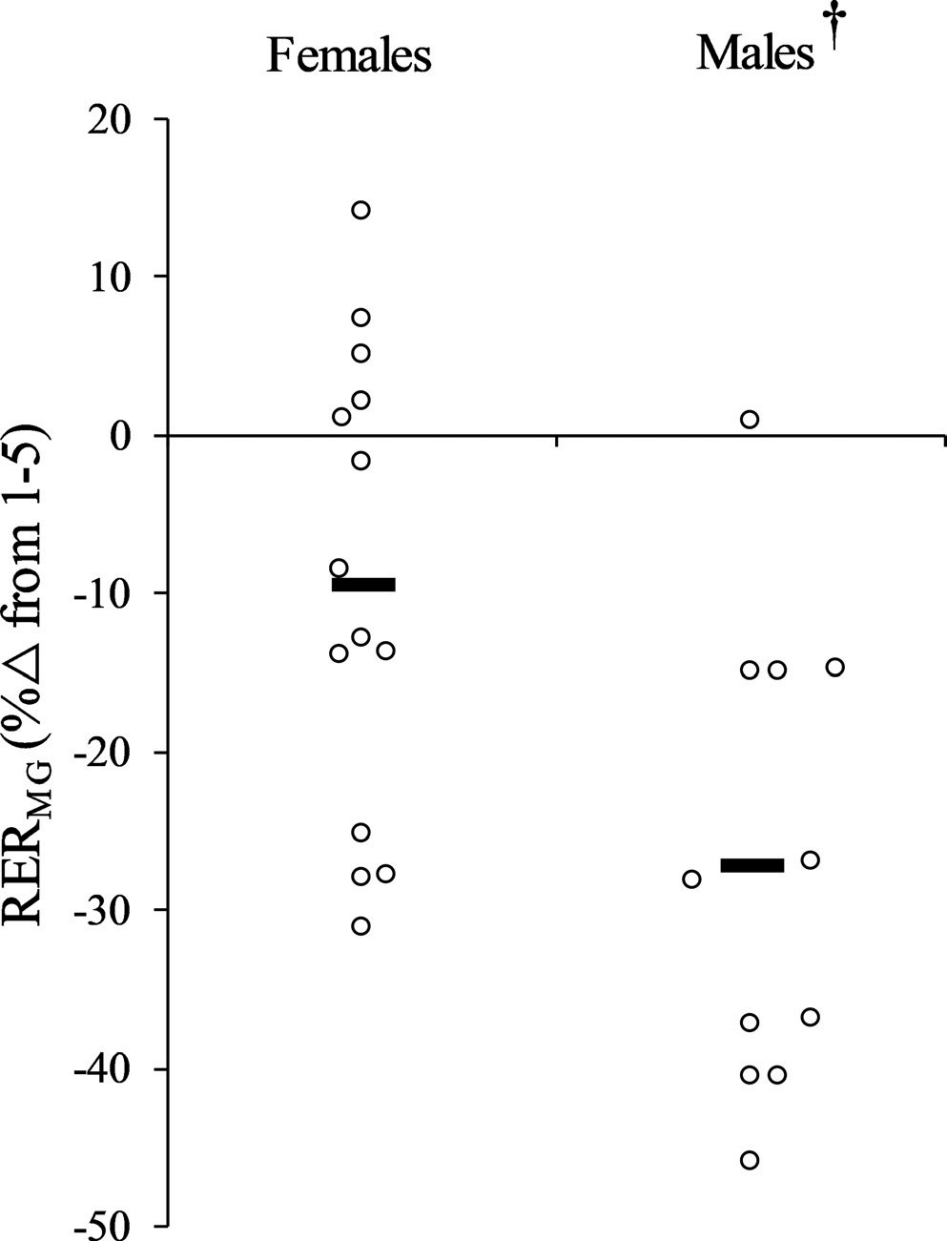
Individual responses and group means (bars) for rate of electromyography rise of the medial gastrocnemius (RER_MG_) during contractions 116–120 of the dynamic fatigue protocol. ^†^Significantly (*p* = 0.038) greater reduction in males compared to females

Figure 2 displays individual relative changes for power during the isotonic fatigue protocol. Power decreased during repetitions 56–60 and 116–120 (*p* < 0.001, $\eta_{p}^{2}$ = 0.669) with no differences between sexes (sex × time, *p* = 0.941, $\eta_{p}^{2}$ = 0.000; sex effect, *p* = 0.832, $\eta_{p}^{2}$ = 0.002). OPT_V_ decreased during repetitions 56–60 (males = 18.05 ± 5.12%; females = 17.03 ± 8.32%) and 116–120 (males = 28.00 ± 6.16%; females = 25.75 ± 13.85%) (*p* < 0.001, $\eta_{p}^{2}$ = 0.572) with no differences between sexes (sex × time, *p* = 0.712, $\eta_{p}^{2}$ = 0.006; sex effect, *p* = 0.618, $\eta_{p}^{2}$ = 0.011). OPT_T_ decreased during repetitions 56–60 (males = 18.98 ± 4.87%; females = 19.70 ± 10.93%) and 116–120 (males = 26.58 ± 4.41%; females = 28.71 ± 13.02%) (*p* < 0.001, $\eta_{p}^{2}$ = 0.572) with no differences between sexes (sex × time, *p* = 0.637, $\eta_{p}^{2}$ = 0.009; sex effect, *p* = 0.679, $\eta_{p}^{2}$ = 0.007). RVD decreased during repetitions 56–60 (males = 29.31 ± 5.89%; females = 28.59 ± 13.62%) and 116–120 (males = 43.42 ± 7.33%; females = 39.30 ± 15.50%) (*p* < 0.001, $\eta_{p}^{2}$ = 0.694) with no differences between sexes (sex × time, *p* = 0.322, $\eta_{p}^{2}$ = 0.041; sex effect, *p* = 0.576, $\eta_{p}^{2}$ = 0.013). RTD decreased during repetitions 56–60 (males = 22.22 ± 14.35%; females = 18.34 ± 15.92%), but did not decrease again during repetitions 116–120 (males = 29.29 ± 23.98%; females = 16.99 ± 24.94%) (*p* = 0.488, $\eta_{p}^{2}$ = 0.020). No differences between sexes were found (sex × time, *p* = 0.310, $\eta_{p}^{2}$ = 0.043; sex effect, *p* = 0.254, $\eta_{p}^{2}$ = 0.054). Figure 3 displays individual relative changes for RER_MG_ during dynamic fatigue. RER_MG_ was decreased more in males compared to females during repetitions 56–60 and 116–120 (sex effect, *p* = 0.038, $\eta_{p}^{2}$ = 0.174). This difference was similar at both time points (sex × time, *p* = 0.170, $\eta_{p}^{2}$ = 0.080; time effect, *p* = 0.614, $\eta_{p}^{2}$ = 0.011). RER_SOL_ decreased during repetitions 56–60 (males = 16.73 ± 19.03%; females = 21.44 ± 20.76%), but did not decrease again during repetitions 116–120 (males = 21.88 ± 19.70%; females = 15.39 ± 16.11%) (*p* = 0.898, $\eta_{p}^{2}$ = 0.001). No differences between sexes were found (sex × time, *p* = 0.119, $\eta_{p}^{2}$ = 0.103; sex effect, *p* = 0.897, $\eta_{p}^{2}$ = 0.001). ROM did not decrease during repetitions 56–60 (males = 2.18 ± 2.36%; females = 1.09 ± 2.17%) but did during repetitions 116–120 (males = 5.84 ± 4.35%; females = 4.49 ± 7.17%) (*p* = 0.002, $\eta_{p}^{2}$ = 0.343) with no differences between sexes (sex × time, *p* = 0.899, $\eta_{p}^{2}$ = 0.001; sex effect, *p* = 0.421, $\eta_{p}^{2}$ = 0.027). POS_PP_ did not change during repetitions 56–60 (males = 5.12 ± 6.50%; females = 1.77 ± 2.93%) or 116–120 (males = 5.64 ± 9.86%; females = 6.25 ± 9.62%) (*p* = 0.184, $\eta_{p}^{2}$ = 0.072) and this was similar for both sexes (sex × time, *p* = 0.292, $\eta_{p}^{2}$ = 0.046; sex effect, *p* = 0.578, $\eta_{p}^{2}$ = 0.013).

### 3.3 Fatigue after isometric exercise

**FIGURE 4:**
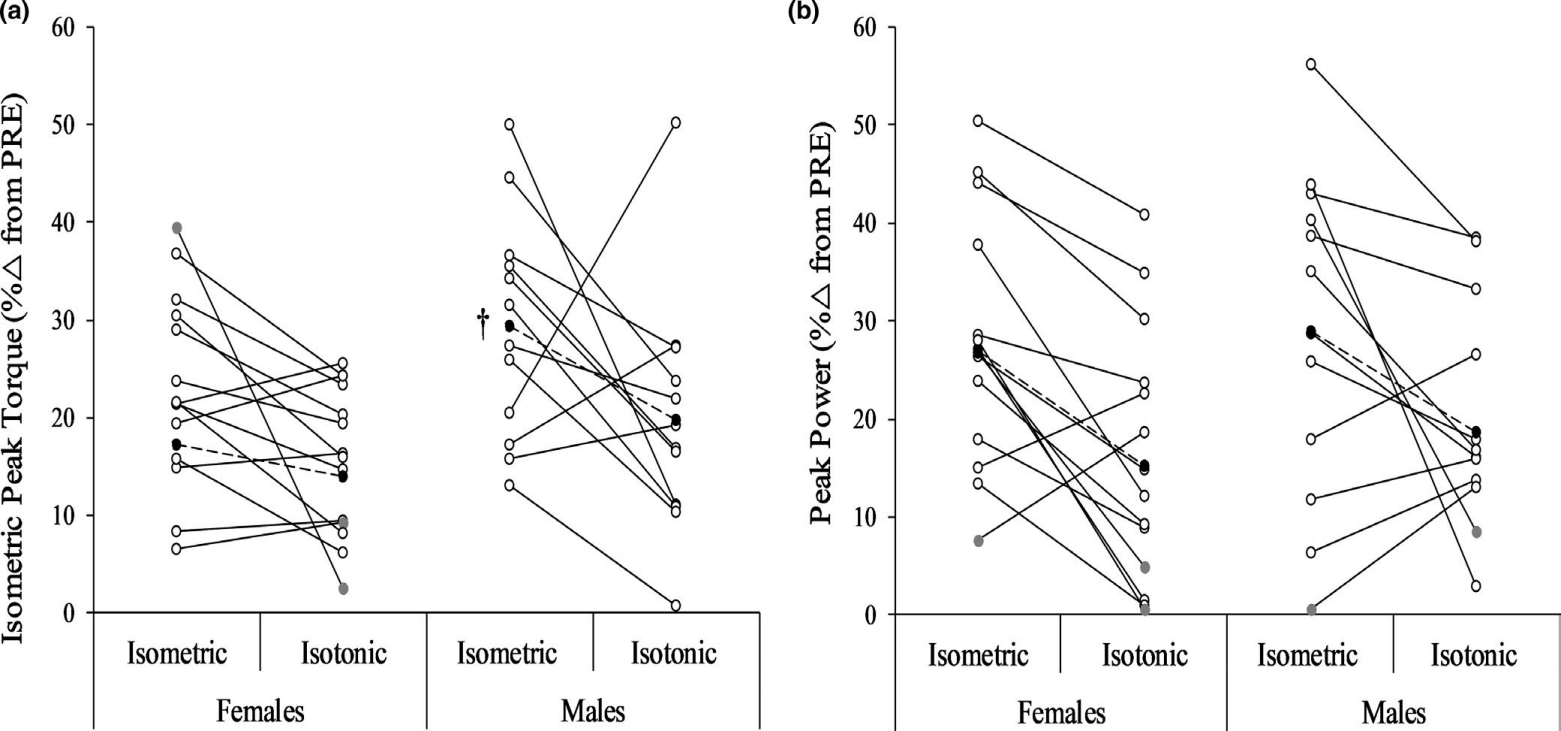
Individual responses and group means (black filled circles) for isometric peak torque (a) and peak power (b) immediately after both fatiguing protocols. Grey filled circles indicate a positive percent change for an individual. ^†^Near significantly (*p* = 0.061) greater reduction in males compared to females for the isometric protocol only

Absolute and relative change values for all performance variables from the isometric fatigue visit are provided in Table 2. Figure 4 displays individual relative changes in isometric peak torque and isotonic power after the isometric protocol. Although not significant, a nearly large effect size was demonstrated for the fatigue‐induced decrease in isometric strength (*p* = 0.061; *d* = 0.77) due to relative decreases tending to be greater in males. (Table 2 and Figure 4). In consideration of the potential influence by the +39% response for one female, an analysis was performed with this female participant excluded and the result was unchanged (*p* = 0.073; *d* = 0.75). The change in isotonic power was similar between sexes (males = 28.96 ± 17.17%; females = 26.94 ± 14.94%) (*p* = 0.752, *d* = 0.12). RPE was also similar in males (8.00 ± 1.86) and females (8.64 ± 1.22) after isometric exercise (*p* = 0.319, *d* = 0.41).

### 3.4 Fatigue after isotonic exercise

Absolute and relative change values for all performance variables from the isotonic visit are provided in Table 3. Figure 4 displays individual relative changes in isometric peak torque and isotonic power after the isotonic protocol. Relative changes in isometric peak torque (*p* = 0.216, *d* = 0.50) and isotonic power (*p* = 0.533, *d* = 0.24) were similar between sexes (Table 3 and Figure 4). RPE was also similar in males (7.67 ± 1.87) and females (8.71 ± 1.38) after isotonic exercise (*p* = 0.319, *d* = 0.41).

### 3.5 Recovery following isometric exercise

**FIGURE 5:**
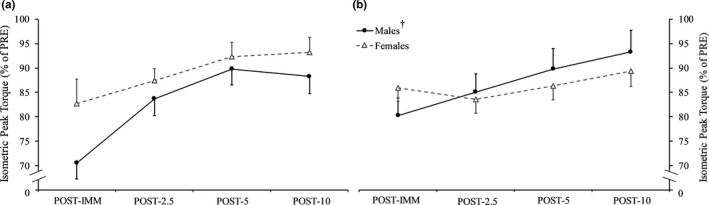
Isometric peak torque immediately (POST‐IMM) as well as 2.5 (POST‐2.5), 5 (POST‐5), and 10 (POST‐10) min after isometric (a) and isotonic (b) fatigue. ^†^Near significantly greater increase throughout recovery after isometric (*p* = 0.053) and isotonic (*p* = 0.072) fatigue in males compared to females

**FIGURE 6:**
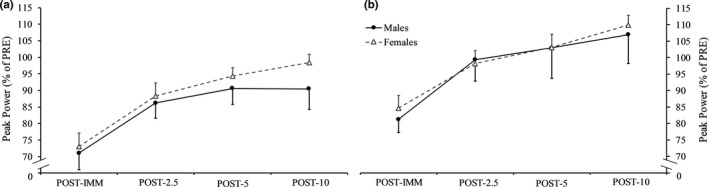
Peak power immediately (POST‐IMM) as well as at 2.5 (POST‐2.5), 5 (POST‐5), and 10 (POST‐10) min after isometric (a) and isotonic (b) fatigue

**FIGURE 7:**
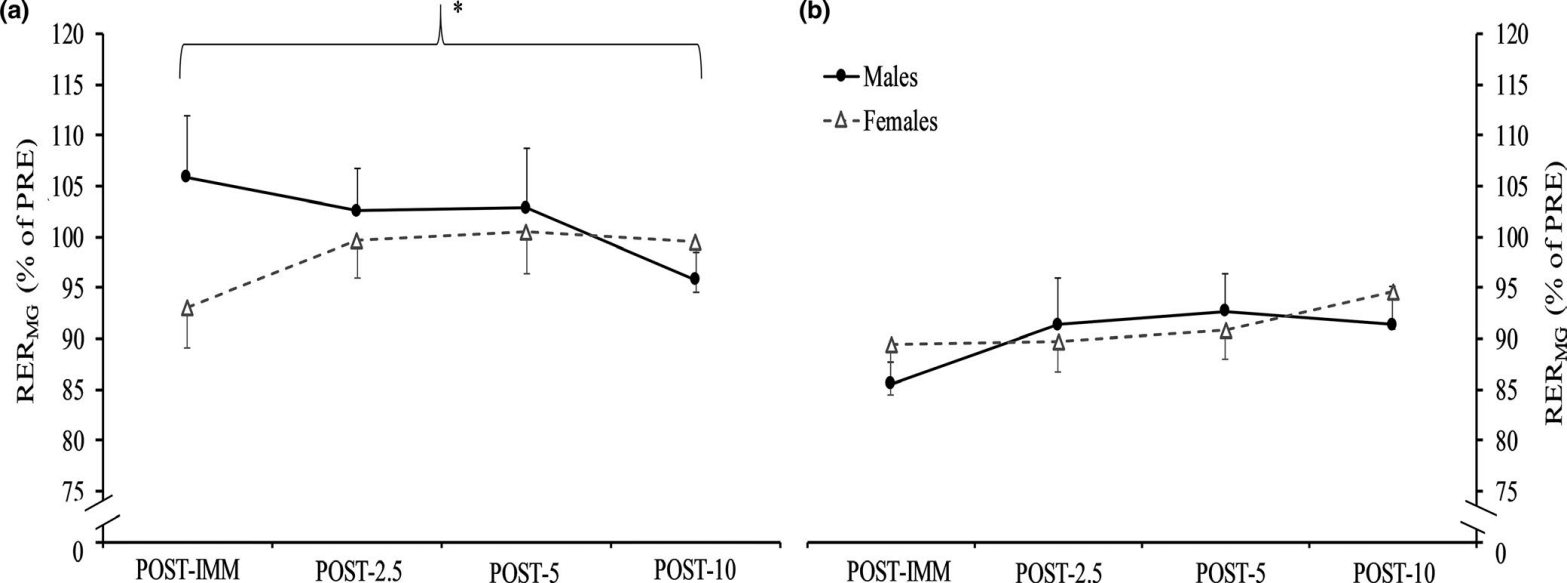
Recovery for rate of EMG rise of the medial gastrocnemius (RER_MG_) immediately (POST‐IMM) as well as at 2.5 (POST‐2.5), 5 (POST‐5), and 10 (POST‐10) min after isometric (A) and isotonic (B) fatigue. *Significant (*p* = 0.043) main effect for sex where females showed greater relative increases throughout recovery compared to males only after the isometric protocol

Figures 5a and 6a display the responses for isometric peak torque and isotonic power, respectively, throughout recovery after isometric fatigue. There were no sex × time interactions for any dependent variables (*p* = 0.726–0.087, $\eta_{p}^{2}$ = 0.015–0.101). For isometric peak torque, there was a nearly significant main effect for sex (*p* = 0.053, $\eta_{p}^{2}$ = 0.147) with males tending to show greater relative increases throughout recovery. We also ran this analysis while excluding the female participant who demonstrated a +39% response for isometric peak torque. The result was relatively similar, albeit a reduction towards a medium sized effect was shown (*p* = 0.092, $\eta_{p}^{2}$ = 0.119). RER_MG_ demonstrated a main effect for sex (*p* = 0.043, $\eta_{p}^{2}$ = 0.166) as relative increases were greater for females throughout recovery (8.79% vs. −3.24%) (Figure 7). No other main effects for sex were present (*p* = 0.981–0.215, $\eta_{p}^{2}$ = 0.000–0.063). With groups collapsed, relative increases in isometric peak torque and isotonic power occurred at POST‐5 compared to POST‐2.5 (*p* < 0.001, 21.85% vs. 13.93% and *p* = 0.008, 32.12% vs. 23.27%, respectively) and then remained steady (*p* > 0.05).

### 3.6 Recovery following isotonic exercise

Figures 5b and 6b display the responses for isometric peak torque and power, respectively, throughout recovery after isotonic fatigue. There were no sex × time interactions (*p* = 0.744–0.105, $\eta_{p}^{2}$ = 0.008–0.095) or main effects for sex (*p* = 0.728–0.290, $\eta_{p}^{2}$ = 0.005–0.047) for any dependent variables. Isometric peak torque nearly demonstrated a main effect for sex (*p* = 0.072; $\eta_{p}^{2}$ = 0.129). With groups collapsed, a relative increase in isometric peak torque only occurred at POST‐10 compared to POST‐2.5 (*p* < 0.001, 11.42% vs. 2.76%) and was similar between POST‐5 and POST‐2.5 (*p* = 0.091) as well as POST‐10 and POST‐5 (*p* = 0.071). Relative increases in isotonic power was similar between POST‐5 and POST‐2.5 (*p* = 0.127), but greater at POST‐10 compared to POST‐5 (*p* = 0.003, 31.22% vs. 24.07%).

### 3.7 Associations

**FIGURE 8:**
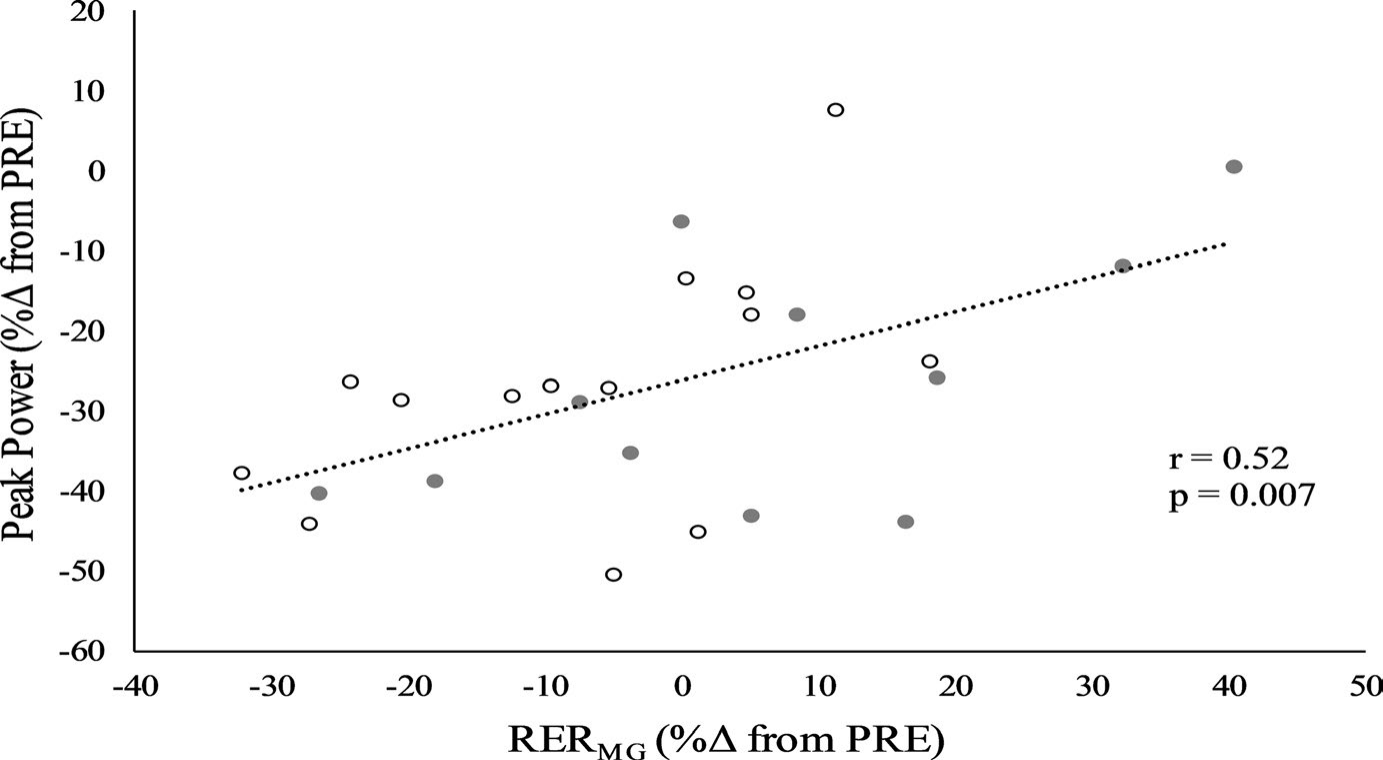
Scatterplot with line of best fit for the relationship between relative changes in power and rate of electromyography rise of the medial gastrocnemius (RER_MG_) immediately after the isometric protocol. Filled and unfilled circles represent males and females, respectively

Figure 8 displays the relationship between relative changes from baseline in power and RER_MG_ immediately after the isometric protocol. OPT_V_, OPT_T_, and RVD exhibited strong correlations (*p* < 0.001) with the relative change in power that were similar after isometric (*r* = 0.94, *r* = 0.96, *r* = 0.96, respectively) and dynamic (*r* = 0.96, *r* = 0.98, *r* = 0.95, respectively) fatigue. RTD and RER_MG_ were moderately correlated with relative changes in power after isometric (*r* = 0.53, *p* = 0.005; *r* = 0.52, *p* = 0.007, respectively) but not dynamic fatigue (*r* = 0.19, *p* = 0.338; *r* = –0.32, *p* = 0.879, respectively). OPT_V_, OPT_T_, and RVD remained strongly correlated with relative changes in power throughout recovery (*r* ≥ 0.90, *p* < 0.001), while the only other correlate was RTD at POST‐10 (*r* = 0.60, *p* = 0.001). Isometric peak torque was not correlated with changes in power at any timepoint (*r* = 0.02–0.31, *p* = 0.898–0.132).

## 4 DISCUSSION

The purpose of this study was to investigate potential sex differences in the fatigue‐ and recovery‐induced responses of power and isometric strength, as well as select dynamic contractile parameters after isometric and isotonic PF contractions. While select studies have examined sex differences in the fatigue‐induced response of power, to the best of our knowledge, this is the first study to examine the fatigue‐related response of power elicited by isometric *and* isotonic muscle contractions. Furthermore, the investigation of the recovery response for power and isometric strength was a novel aspect of the present study. Our findings indicated that the fatigue‐induced changes in dynamic contractile parameters and isometric strength were similar between males and females after both, isometric and isotonic exercise. One exception was RER which demonstrated a unique fatigue and recovery response between sexes but this was true only for the MG. Otherwise, recovery of muscle function was similar for males and females after both fatiguing modalities. It was also shown that relative changes in isometric strength failed to correlate with changes in power after fatigue or during recovery. Finally, some correlates (i.e., RTD, RER_MG_) of the relative change in power were dependent upon the type of fatiguing exercise.

The present study found no sex differences in the response of power or isometric strength, regardless of fatiguing modality. Our findings are similar to previous studies (Senefeld et al., 2013; Senefeld et al., 2018; Yoon et al., 2015) that found power to decrease similarly between sexes after fatiguing dynamic contractions of the knee extensors and elbow flexors. In contrast, Lanning et al. (2017) demonstrated reduced fatigability of power in females after isotonic contractions of the same muscle group tested in the present study (i.e., PFs). This discrepancy is likely due to the difference in the number of contractions performed. The present study involved 120 contractions, whereas Lanning et al. (2017) used 200 contractions. As suggested by Lanning et al. (2017), sex differences in dynamic fatigability of the PFs may only be induced by relatively “extreme” protocols. Studies demonstrating greater fatigability in males for isometric strength have shown this primarily for the knee extensors (Senefeld et al., 2013, 2018) and elbow flexors (Hunter et al., 2006; Maughan et al., 1986), as well as other muscle groups. There is limited evidence for the PFs, but Hatzikotoulas et al. (2004) showed similar findings to the present study, which may suggest that sex differences in fatigability are not substantial for this muscle group. However, in partial support of our hypothesis, the relatively large effect size demonstrated for isometric strength is noteworthy and may indicate a tendency for males to be more fatigable. In accordance with Senefeld et al. (2018), sex differences in fatigue may be primarily demonstrated through isometric strength as opposed to power. Given these mixed findings for lower‐body muscles, greater diversity with respect to the locomotive muscles investigated is needed in order to understand which lower‐body muscles may exhibit greater sex differences in fatigability, and thus be more influential in limiting performance.

In support of our hypothesis, there were no sex differences in fatigability of any dynamic contractile parameters, which was not necessarily surprising since power decreased similarly between sexes. Senefeld et al. (2018) also found similar fatigue related changes in velocity, torque, and RVD of the knee extensors after dynamic fatigue. Lanning et al. (2017) found OPT_T_ of the PFs to be more fatigue resistant in females, but differences between subject characteristics or the number of contractions performed may explain this discrepancy. It is important to note that nearly all measures of muscle function were reduced more after isometric compared to dynamic fatigue. This is likely a result of the maximal intensity associated with the isometric task which should have required greater motor unit activation as compared to the submaximal isotonic protocol. Fatigue induced by dynamic contractions is thought to primarily have a peripheral origin (Babault et al., 2006; Cheng & Rice, 2005). Indeed, Senefeld et al. (2018) recently demonstrated that males exhibited greater peripheral fatigue compared to females after fatiguing isotonic contractions for the knee extensors. However, in the present study, RER_MG_ was diminished more in males compared to females *during* isotonic contractions. This finding may reflect a greater impairment in rapid CNS activation of the MG in males (Klass et al., 2008; Van Cutsem et al., 1998), and not necessarily peripheral changes. However, the potential influence of sarcolemmal changes on EMG‐derived parameters cannot be ruled out (Dimitrova & Dimitrov, 2003). Interestingly, there was no difference between sexes immediately after (~6 s) the isotonic contractions which suggests rapid recovery for rate of muscle activation in males upon immediately ceasing exercise. As expected, similar to Lanning et al. (2017), percent changes in OPT_T_, OPT_V_, and RVD, were associated with changes in power. An interesting finding was that RTD and RER_MG_ (see Figure 8) were correlated with changes in power only after isometric fatigue. This could be indicative of a distinct etiology of fatigue induced by the two modalities, but this cannot be confirmed and this finding is likely confounded by the greater amount of fatigue elicited by the isometric protocol.

As can be seen in Figures 5, 6, 7, the greatest rate of recovery occurred within the first 2.5 min after fatigue. Although commonly examined recovery timepoints were used in the current study, future research may want to take this into consideration through the use of earlier recovery timepoints (e.g., 1 min). All muscle function variables recovered similarly in males and females for both fatiguing modalities. The near significantly greater recovery of isometric strength in males after both fatiguing protocols was likely influenced by the relatively greater decrease (see Figure 4) in isometric strength compared to females. These findings contrast our hypothesis and the findings of Senefeld et al. (2018) who showed greater recovery in females for isometric strength after isometric and isotonic knee extensor exercise. Collectively, this likely reflects muscle‐specific differences in the recovery response between sexes, but more studies specifying the fatigue and recovery response are needed especially for lower‐body muscles. While relative increases in RER_MG_ were greater throughout recovery in females after isometric exercise, this should be interpreted with caution as this finding was almost certainly influenced by RER_MG_ being largely unaffected in males compared to females (6% vs. −7%) immediately after isometric exercise. The latter in conjunction with the RER_MG_ findings *during* isotonic exercise may suggest unique responses for the PFs depending on the type of fatiguing exercise. It is worthwhile for future work to investigate the potential for sex differences in the fatigue‐related responses of the MG and SOL. Isometric strength and isotonic power recovered differently after isotonic exercise as the latter showed a quicker rate of recovery. As suggested by Krüger et al. (2019) and demonstrated by Akagi et al. (2020), fatigue‐induced changes in isometric strength and power should not be considered interchangeable as they follow different timelines and likely reflect at least some unique physiological processes. Similar to Akagi et al. (2020), relative changes in isometric strength were not correlated with power at any timepoint. As expected, percent changes in OPT_T_, OPT_V_, and RVD were strongly correlated with the change in power during recovery after both fatiguing protocols, but RTD and RER_MG_ were moderately correlated with changes in power only after isometric fatigue. While some caution is needed when interpreting these correlational findings, these task‐specific associations for RTD and RER_MG_ may reflect unique physiological alterations during recovery after isometric and dynamic fatigue, as was recently reported (Senefeld et al., 2018).

There were a few limitations associated with the present study. There was marked within‐group variability in the fatigue‐induced changes for either exercise protocol. Standardizing the relative degree of fatigue (i.e., % decrease in power) would have decreased variability, but this was not possible since different modalities of fatiguing exercise were examined. As mentioned previously, it is apparent that the response of power and isometric strength to fatiguing exercise is different. A surprising finding was that power was greater at baseline during the isometric protocol compared to the dynamic protocol. The reason for this is unclear since the order of the protocols was randomized and a familiarization visit was implemented, but it is possible the relatively large range of days between visits (2–7 days) may have been an influential factor. Additionally, while the current study focused on relevant mechanical contributors to fatigue‐ and recovery‐related changes in power, details regarding the etiology of neuromuscular changes were not investigated. The inclusion of electrical stimulation during voluntary contractions and at rest would have provided further understanding of the peripheral and central mechanisms associated with our findings.

## 5 CONCLUSIONS

Sex differences in the fatigability of isometric strength and, to a lesser extent, power have been widely studied in various muscle groups, but evidence is scant for the plantar flexors. The current study comprehensively investigated muscle function through strength and power testing and incorporated a novel approach by independently assessing the fatigue *and* recovery response for these parameters after isometric and isotonic exercise. It was shown that sex differences in fatigability were not present for isotonic power and other dynamic contractile properties after isometric or isotonic exercise. Similarly, fatigue‐induced decrements in isometric strength between sexes were not significantly different, but males tended to express greater relative reductions. A sex‐dependent etiology of fatigue, as suggested by a reduced rate of muscle activation in males only, may have been present after dynamic contractions, but this could not be verified given the present methodology. Similar to fatigability, recovery of muscle function was also similar between sexes. In support of recent studies (Akagi et al., 2020; Krüger et al., 2019), fatigue‐ and recovery‐related responses in maximal strength did not correspond with changes in power, thus is it important for future work to consider these measures and their physiological contributors independently. The present work indicates that sex differences in fatigability for the plantar flexors may not be as prominent as other commonly examined muscle groups. Given the important role of the plantar flexors for locomotion, future work is needed to reveal the relevant considerations (i.e., contraction type, intensity, etc.) related to the specific, if any, sex differences that may exist for this muscle group.

## CONFLICT OF INTEREST

The authors have no conflicts of interest.

## AUTHOR CONTRIBUTIONS

Phuong L. Ha: Conception and Design, Data Acquisition, Data Processing, Statistical Analysis, Data Interpretation, and Writing—original draft and revisions; Benjamin E. Dalton: Data Acquisition, Data Processing, Data Interpretation, and Writing—revisions; Michaela G. Alesi: Data Acquisition, Data Processing, and Writing—revisions; Tyler M. Smith: Data Acquisition, Data Processing, and Writing—review and editing; Trisha A. VanDusseldorp: Conception and Design, and Writing—review and editing; Yuri Feito: Conception and Design, and Writing—review and editing; Garrett M. Hester: Supervision, Conception and Design, Statistical Analysis, Data Interpretation, and Writing—original draft and revisions. All authors approved the final version of the manuscript.
